# Evaluation of Innovative Dried Purée from Jerusalem Artichoke—In Vitro Studies of Its Physicochemical and Health-Promoting Properties

**DOI:** 10.3390/molecules26092644

**Published:** 2021-04-30

**Authors:** Jan Oszmiański, Sabina Lachowicz, Paulina Nowicka, Paweł Rubiński, Tomasz Cebulak

**Affiliations:** 1 Department of Fruit, Vegetables and Nutraceutical Technology, Wrocław University of Environmental and Life Science, Chełmońskiego 37, 51-630 Wrocław, Poland; jan.oszmianski@upwr.edu.pl (J.O.); paulina.nowicka@upwr.edu.pl (P.N.); 2 Department of Fermentation and Cereals Technology, Wrocław University of Environmental and Life Science, Chełmońskiego 37, 51-630 Wrocław, Poland; 3 Calisia University, Nowy Świat 4, 62-800 Kalisz, Poland; pawel.rubinski@interia.pl; 4 Department of Food Technology and Human Nutrition, University of Rzeszow, Zelwerowicza 4, 35-601 Rzeszów, Poland; tomcebulak@gmail.com

**Keywords:** functional food, innovative food, drying, natural food, *Helianthus tuberosus*, pro-healthy properties

## Abstract

The present study aimed to evaluate the effect of Jerusalem artichoke processing methods and drying methods (freeze drying, sublimation drying, vacuum drying) on the basic physicochemical parameters, profiles and contents of sugars and polyphenolic compounds, and health-promoting properties (antioxidant activity, inhibition of the activities of α-amylase, α-glucosidase, and pancreatic lipase) of the produced purée. A total of 25 polyphenolic compounds belonging to hydroxycinnamic phenolic acids (LC-PDA-MS-QTof) were detected in Jerusalem artichoke purée. Their average content in the raw material was at 820 mg/100 g dm (UPLC-PDA-FL) and was 2.7 times higher than in the cooked material. The chemical composition and the health-promoting value of the purées were affected by the drying method, with the most beneficial values of the evaluated parameters obtained upon freeze drying. Vacuum drying could offer an alternative to freeze drying, as both methods ensured relatively comparable values of the assessed parameters.

## 1. Introduction

The Jerusalem artichoke (*Helianthus tuberosus*; (JA)) is a species of sunflower from the genus Helianthus, belonging to the family Asteraceae, and derived from the North America. In Europe, it has been cultivated since the 17th century [1,2]. It is characterized by a fast growth rate, is tolerant to droughts, salinity, and frost, and is resistant to diseases and pests [2,3]. Due to its valuable chemical composition and scientifically proven health-promoting properties, JA has spurred a growing interest as an edible plant [4]. Its tubers contain ca. 80% of water, 2% of protein, and ca. 20% of carbohydrates, ca. 90% of which are represented by inulin [2]. JA is also valuable considering its bioactive compounds, such as, e.g., polyphenolic compounds, including phenolic acids, which exhibit strong antioxidant properties, and has also been confirmed to elicit antiviral, antibacterial, anti-inflammatory, and anti-carcinogenic effects [1,5]. In turn, as a prebiotic and soluble dietary fiber, inulin contained in JA tubers and stalks (considered to be its richest sources) ensures a hypoglycemic effect in diabetes treatment. In the gastrointestinal tract, inulin undergoes fermentation by the gut microbiota, affecting the state of eubiosis. In addition, it contributes to the increased availability of such minerals as Fe, Mg, and Ca, and influences lipid metabolism [1,6,7]. Furthermore, JA improves immunity and concentration, alleviates stress, and eliminates toxic metabolites from the body [8]. Its main applications include the production of inulin [9], feedstuff, fructose syrup, flour, French fries, biochemical materials, and bioethanol [10,11,12]. JA tubers processed with various cooking methods were evaluated for their sensory profiles [12]. Thus, taking into account their beneficial effects of providing valuable substances, it is necessary to develop a product with the lowest possible losses of these valuable substances and high storage stability. 

Considering the above, we proposed JA purée preserved with a properly selected drying method. The use of the drying process will enable the preservation of the product, making it available all year round, and not only in the maturity period. The most common drying method is convective drying due to its low cost and relatively high efficiency [13]. In turn, sublimation drying (SD) requires high temperatures, is relatively long, which in turn leads to large losses of compounds valuable to the human body, resulting from the high access of oxygen [13]. On the other hand, freeze drying (FD) is the best method to obtain products with the lowest thermal degradation of bioactive compounds during water removal; however, it is relatively costly. Hence, vacuum drying (VD) can be an alternative to SD because it offers a shorter drying time, due to the reduced pressure, and heat supply by conduction [14]. In addition, it allows temperature control, which can reduce the thermal degradation of thermolabile compounds, such as polyphenolic compounds, and is relatively economical. However, different drying processes can affect the quality and induce different positive or negative changes in the finished product. Therefore, it is important to monitor these changes depending on the product being dried. Furthermore, the impact of the technological treatment and changes induced by drying on the content of inulin and polyphenols, and health-promoting values in the innovative dried purée from JA (raw and cooked tubers) has not been studied so far. Considering the above, the present study aimed to evaluate the effect of Jerusalem artichoke processing and drying methods (freeze drying, sublimation drying, vacuum drying) based on the analysis of physicochemical parameters, profiles and contents of sugars and polyphenolic compounds, and health-promoting properties (antioxidant, anti-diabetic, and anti-obesity activity) of the produced purée.

## 2. Results

### 2.1. Chemical Parameters

#### 2.1.1. Basic Chemical Parameters

The results of analyses of six variants of dry purées obtained from fresh and cooked JA are presented in Table 1. Both the drying methods and the purée preparation technology statistically significantly affected the ash and pectin contents, while they had no significant effect on the dry matter content, total acidity, and pH (*p* < 0.05). The FD products showed a higher content of dry matter, ash, and pectins. In turn, the lowest contents of pectin, ash, and dry matter were obtained in the products after SD. Therefore, this method was proved the most advantageous for the preparation of the innovative dried JA purée; however, due to its high costs, the VD can be used as an alternative. Taking into account the JA preparation technology, the greatest differences were noted in SD products, where the contents of dry matter and ash were 5% and 9% higher in the purée prepared from cooked JA, while pectin content was 41% higher in the purée made of fresh material. In the case of pectins, after VD and FD, their content was 12% and 28% lower in the purée made from the cooked material than from the raw one. This is because cooking causes plant tissues to break down into individual cells and pectins to leach out. This phenomenon is also characteristic of potatoes and JA, but it is not observed when cooking root vegetables due to their thicker and harder cell membranes [15]. In addition, pectin is a form of soluble fiber that helps prevent cardiovascular diseases, diabetes, and obesity [16]. Therefore, a purée prepared from raw JA will be a more desirable product, especially when designing products dedicated to obese and diabetic patients.

#### 2.1.2. Determination of Sugar Changes

Table 1 presents the results of sugar content determination by ultra-efficient liquid chromatography coupled with an ELSD detector. The assessed material contained three types of sugars, i.e., inulin (accounting for 95.8% of total sugars on average from all analyzed sample) > sucrose (3.7%) > fructose in trace amounts (0.5%). The highest amount of total sugar was ranged from 41.4 g/100 g in sample made of raw material after the VD drying method to 45.3 g/100 g in sample made of raw material after the FD drying method. In turn, the highest content of inulin was determined in the purée from raw JA after FD (43.3 g/100 g), and the lowest one in the purée from raw JA after VD (38.9 g/100 g). Similar results concerning inulin content in the material subjected to FD were obtained by Michalska-Ciechanowska et al. [17] and Cieslik et al. [18]. In the case of fructose, it has been noted that the higher the inulin content was, the lower was the fructose content. This means that inulin has not been hydrolyzed to fructose, which usually occurs by an acid or by inulinase [19]. The authors state that JA contains the highest amounts of inulin during the harvest period from October to December, and that in the remaining months, inulin is hydrolyzed to a simple sugar [19]. However, it was noted that the purée made of raw JA contained from 29% to 78% more fructose after the FD and VD drying method, respectively, compared to the purée made of cooked JA. Thus, this may be related to inulin hydrolysis. According to Böhm et al. [20] the 1 h heat treatment at 100 to 135 °C after the acid hydrolysis of chicory inulin did not affect its degradation, which may also explain the slight differences between the purées prepared from the cooked and raw JA.

#### 2.1.3. Determination of Polyphenolic Compounds

The detailed identification of polyphenolic compounds in dried JA purée made of raw and cooked material dried with all drying methods using LC-PDA-MS-QTof and UPLC-PDA-FL showed the presence of 25 compounds, all of which belonged to the class of hydroxycinnamic phenolic acids (Table 2). Similar results were obtained by Kapusta et al. [21] and Michalska-Ciechanowska et al. [17]. The obtained results indicate that the drying method used had a significant (*p* < 0.05) effect on the content of polyphenolic compounds. The highest total content of polyphenols, reaching 923.5 mg/100 g d.m., was found in the purée made of raw JA after FD. It was about five times higher compared to the content of polyphenolic compounds determined by Kapusta et al. [21]. Depending on the drying method used, the result obtained after FD was 21% (made of cooked material) and 15% (made of raw material) higher compared to the results obtained after SD and VD drying, respectively. According to Michalska et al. [14], the drying method had a significant impact on the final content of bioactive compounds in the finished product; hence, it is essential to select the appropriate drying method that would allow the maintenance of a relatively high content of the tested compounds. In the present study, the content of polyphenols was also statistically significantly affected by the technological treatment of JA. The purée made of cooked JA tubers contained 62% for FD, 69% for SD, and 60% for VD less bioactive compounds than the purée obtained from the unprocessed raw material dried by FD, SD, and VD, respectively. Similar results were reported in the study by Laib and Barkat [22], in which the content of polyphenolic compounds was much lower in cooked potatoes than those that were not heat-treated. This is probably due to the fact that these compounds are thermolabile, thus were destroyed and leached out to the solution. 

3-*O*-caffeoylquinic acid (with main ion at *m*/*z* 353) and 3,5-di-*O*-caffeoylquinic acid (with main ion at *m*/*z* 515) were the major compounds [17] and, on average, accounted for 18% (from 16% of FDR to 24% of FDC) and 11% (from 8% of SDC to 13% of FDR) of total phenolic hydroxycinnamic acids, respectively. Their contents were also significantly dependent on the drying method and technological treatment, with the highest ones determined in the purées prepared from raw JA after FD, while the lowest ones were in the purée made of cooked JA after SD. During the peeling of Jerusalem artichoke, sulfur dioxide was added to protect the color. The UPLC-PDA chromatograms (Supplementary Materials Figure S1) of purées made of raw JA tuber revealed additional peaks of compounds, which were identified as derivatives of quinones, i.e., phenolic acid oxidation products in combination with a sulfite molecule. Such a combination with quinone sulfite was noted in caffeoylquinic acid (with main ion at m/z 415), compounds, whose fragmentation ions were found at *m*/*z* 387, 258, 191, 179, and 161, and in dicaffeoylquinic acid compounds (with main ion at *m*/*z* 577) whose fragmentation ions were found at *m*/*z* 415, 387, 258, 191, 179, and 161. The contribution of derivatives of quinones on the total polyphenolic compound concentration ranged from 30% for FDR to 34% for SDR. In turn, these compounds were not identified in the purées made of cooked JA tubers. They were probably washed out from the root surface by the water solutions in which they were cooked. In addition, the high cooking temperature contributed to the inactivation of enzymes that could cause the oxidation of phenolic acids to quinones. The use of sulfur dioxide as a preservative had a significant impact on the protection of bioactive compounds in the purées made of raw rather than cooked material. Similar observations regarding the protection of polyphenolic compounds were noted upon the use of sulfur dioxide in white wines [23]. In addition, compounds such as caffeoylquinic acids (isomer of chlorogenic acid; with main ion at *m*/*z* 353) and 1,5-dicaffeoylquinic acid (with main ion at *m*/*z* 515) were significantly influenced by cooking, as their contents in the purées were on average 57% and 52% lower, respectively, compared to those determined in the purées made of raw tubers. Similar observations were made by Laib and Barkat [22], who investigated the effect of heat treatment, including cooking, on the content of compounds in potato tubers. The analyzed compounds were probably released during heat treatment because some polyphenols, including phenolic acids, may be associated with non-digestible components of the cellular structure and may be released and/or solubilized during this structure’s damage [22,24]. Moreover, the loss of the analyzed compounds is also strongly affected by their chemical structure, because the cooking process may differently influence compounds classified into one subclass. These losses can be influenced by the hydroxylation pattern, particle size, solubility, polarity, and sugar bonding [25]. Therefore, it is important to monitor changes during the selection of the most advantageous drying technique. The best method to obtain the dried purées turned out to be the freeze drying; however, due to its costs, the vacuum drying seems a fine alternative. In turn, depending on the chemical composition of the finished product, the raw material processing method can be used appropriately when designing new products. Considering the content of bioactive compounds, it is more advisable to produce the dried purée from raw JA tubers, irrespective of the compounds released during processing.

### 2.2. Physical Parameters

#### Color Parameters and Water Activity

The dried purées were found to differ significantly in their color parameters as affected by both the technological treatment and drying method (Table 3). The best in terms of brightest turned out to be the dried purée prepared from raw JA after FD compared to that made of boiled JA. In contrast, the use of VD and SD for product preservation caused 5% and 9% darkening of the purée made of the raw tubers and 5% and 4% darkening of the purée made of the cooked tubers. The a * and b * color parameters of the tested material indicated that JA cooking intensified the green color and darkened the yellow color of the purées, while purées made of the raw material were more yellow with a slight hue of green. Similar dependencies were observed in the measurements of the a* and b* color parameters depending on the drying method used; the FD purées were characterized by a light hue of green and a dark hue of yellow. An opposite tendency was noted in SD products, revealing a darker hue of green and a lighter hue of yellow. The results obtained for the purée after FD were comparable with the color measurement results reported by Antal et al. [2] for JA subjected to FD drying only. Those authors demonstrated a similar dependency; namely that the color of the dried material depended on the drying method used, and thus the brightest products were also obtained after FD [2]. 

The evaluation of the dried purées in terms of water activity (aw) showed statistically significant differences caused by both the drying method and the method of purée preparation (Table 1). The lowest aw, reaching 0.012 for FDC and 0.015 for FDR, was determined for the FD purée, and this value was on average 18 and 11 times lower compared to SD and VD purées, respectively. On the other hand, the a_w_ value determined after VD was two times lower compared to the value determined after SD. According to the results reported by Antal et al. [2], the a_w_ value recorded for freeze-dried JA without technological treatment was seven times higher compared to our study. A lower a_w_ value of dried fruits of Saskatoon berry was also noted after FD, whereas there was a higher value after SD [26]. In turn, regardless of the drying method used, the purées made of raw material were characterized by an on average 40% higher a_w_ value. This can be explained by the slight evaporation of water during the cooking process of [12]. However, regardless of the drying method and preparation technology used, the aw of all dried purées was below the critical level (a_w_ = 0.60). This means that they meet the requirement of a product safe from microbiological spoilage, i.e., from contamination with bacteria and mold, because the a_w_ value above 0.60 may cause microbiological spoilage of the finished product [2].

### 2.3. Pro-Healthy Properties

The study also determined the health-promoting properties of JA preserved using various drying methods. The purées were analyzed in terms of their antioxidant, anti-diabetic (the ability to inhibit α-amylase and α-glucosidase), and anti-obesity properties (the ability to inhibit pancreatic lipase). Finding effective inhibitors of α-amylase and α-glucosidase would allow a delay in sugar absorption and a reduction in postprandial blood glucose. On the other hand, finding an effective inhibitor of pancreatic lipase activity by stimulating the cell membrane permeability would enable the apt functioning of the pancreas as a gland responsible for the proper insulin secretion. Additionally, pancreatic lipase is a key dietary fat-absorbing enzyme responsible for the hydrolysis of triglycerides to 2-monoacylglycerides and free fatty acids that can be absorbed by enterocytes. Its inhibition is used to reduce the rate of dietary fat absorption and, therefore, may offer an alternative approach to treating overweight and obesity [27]. 

The analysis of the antioxidant activity of the studied variants of JA purées showed that the greatest antioxidant effect was obtained in JA dried using the vacuum and sublimation methods (Table 4). It was proved that not only the drying method, but also the type of raw material used for drying, played a significant role in modeling the antioxidant properties of the tested material. Hence, a much better effect in developing the health-promoting properties was obtained by drying fresh than the previously cooked material. 

A slightly different trend was observed in the JA ability to inhibit pancreatic lipase, α-amylase, and α-glucosidase (Table 4). A more effective inhibitor turned out to be SDC. An opposite effect was observed with the vacuum method. It was shown to be the most effective in modeling both anti-diabetic and anti-obesity properties. Moreover, as in the case of antioxidant properties, it appeared more effective to dry fresh raw material than the cooked one. In general, it can be concluded that the produced dried purées were characterized by a high anti-diabetic potential—similar results were obtained for both α-amylase (IC_50_ values from 130 μg/mL to 736 μg/mL) and α-glucosidase (IC_50_ values from 120 μg/mL to 898 µg/mL). A study by Wang et al. [28] has shown that the ability to inhibit α-glucosidase is stimulated by hydroxycinnamic acid derivatives. However, no such trend was observed in the present study. It has been shown that procyanidin polymers can also be involved in enzyme inhibition. This tendency was confirmed by Boath et al. [29], who suggested that fruit extracts rich in procyanidins were effective inhibitors of α-amylase because they had the ability to form tannin–enzyme complexes, which effectively inhibited the hydrolysis of polysaccharides to simple sugars. Other authors have shown that the effective blocking of diabetes-related enzymes may be due to high concentrations of inulin [30].

It should be emphasized, however, that JA was the most effective pancreatic lipase inhibitor. The IC_50_ values determined for this enzyme ranged from 31 µg/mL (air-dried Jerusalem artichoke, previously cooked) to 58 µg/mL (air-dried JA, fresh). The observed trend may again be due to the high concentration of inulin in this product. Recently, it has been proved that inulin effectively prevents the occurrence of obesity and diabetes, i.a., by lowering the blood levels of triglycerides, cholesterol, and glucose [31].

The conducted research has shown that JA is an interesting raw material with a wide spectrum of health-promoting properties that can be modulated in certain ranges by selecting appropriate processing and preservation methods.

## 3. Materials and Methods

### 3.1. Materials 

Acetonitrile, formic acid, methanol, ABTS (2,2′-azinobis(3-ethylbenzothiazoline-6-sulfonic acid), 6-hydroxy-2,5,7,8-tetramethylchroman-2-carboxylic acid (Trolox), 2,4,6-tri(2-pyridyl)-s-triazine (TPTZ), 2,2-Di(4-tert-octylphenyl)-1-picrylhydrazyl (DPPH), methanol, acetic acid, 2,2′-azobis (2-amidino-propane) dihydrochloride (AAPH), fluorescein disodium (FL), potassium persulfate, TPTZ (2,4,6-tripyridyl-1,3,5-triazine), FeCl3, phloroglucinol, 3,5-dinitrosalicylic acid, potassium sodium tartrate tetrahydrate, sodium phosphate monobasic, starch from potato, α-amylase from porcine pancreas (type VI-8), dipotassium hydrogen orthophosphate dihydrogen, p-nitrophenyl-α-D-glucopyranoside, α-glucosidase from Saccharomyces cerevisiae (type I), sugar, and polyphenolic standards were purchased from Sigma-Aldrich (Steinheim, Germany). Acetonitrile for ultra-performance liquid chromatography (UPLC; gradient grade) and ascorbic acid were from Merck (Darmstadt, Germany). 

*Helianthus tuberosus* was harvested at the organic cultivation Gospodarstwo Rolno-Ogrodnicze Marek Strojs in Kalisz (Poland). Jerusalem artichoke (around 10 kg) “Albik” cultivar was collected in August 2020.

### 3.2. Sample Preparation

The production process of dried Jerusalem artichoke purée included 3 main stages:

(a) Peeling: samples were peeled for 3 min at room temperature with the addition of 0.5% sodium metabisulfate dissolved in water (1:1) on a modified vegetable peeler (YATO YG-03087 firmy (TOYA S.A Wrocław Poland).
(b) Preparation of two variants of material for drying, i.e., from fresh and cooked raw material: (i) in the first variant, 1 kg of peeled fresh material was ground in a Thermomix Varoma with the addition of 0.5% sodium metabisulphate (5 min/20 °C); whereas (ii) in the second variant, 1 kg of peeled Jerusalem artichoke was cooked in a basket of the Thermomix in water with the addition of 0.5% sodium metabisulphate (30 min/98 °C); afterward, the water was drained and the cooked sample was ground (5 min/20 °C).
(c) Drying: both cooked and raw samples were dried using three methods: (i) freeze drying (FD)—carried out in a freeze dryer—(Christ Alpha 1–4 LSC; Osterode am Harz, Germany) for 24 h, (ii) sublimation drying (SD)—carried out in a (KC 100/200 Wytwórnia Aparatury Elektronicznej i Medycznej Warszawa) for 8 h at 80 °C, (iii) vacuum drying (VD)—carried out in a VACUCELL 111 ECO LINE vacuum dryer (MMM Medcenter Einrichtungen GmbH, Planegg, Germany) for 8 h at 80 °C.

All drying experiments were performed in duplicate. The samples obtained were milled by a laboratory mill (IKA A.11, Wilmington, NC, USA), and vacuum sealed. The powders were kept in a freezer (−20 °C) until the extracts’ preparation.

### 3.3. Chemical Parameters

#### 3.3.1. Basic Parameters 

The dry matter was determined by mixing the sample with diatomaceous earth, pre-drying, and final drying under reduced pressure. Total acidity was extracted by mixing with water in 100 mL flasks, cooking for 20 min, and cooling for 20 min. Total acidity (TA) was analyzed supernatant (8 mL) by the titration of products with 0.1N NaOH to pH 8.1 and the results were expressed as g malic acid/100 g. TA and pH were determined using an automatic pH titrator system (TitroLine 5000, Xylem Analytics GmbH, Weilheim in Oberbayern, Germany). The soluble solids content, dry matter, titratable acidity, and ash were taken according to European Standards, PN-EN 12143:2000, PN-EN 12145:2001, and PN-EN 12145:2000, PN-EN 450:1998, respectively. Pectin content (g/100 g) was measured according to the Morris method reported by Pijanowski et al. [32]. All measurements were taken three times.

#### 3.3.2. Determination of Sugar by HPLC

An analysis of sugar by the HPLC-ELSD method was performed according to the protocol described by Oszmiański and Lachowicz [33]. Calibration curves (*R*^2^ = 0.9999) were created for glucose, fructose, sorbitol, and sucrose. All data were obtained in triplicate. The results were expressed as g per 100 g dm.

#### 3.3.3. Polyphenolic Compounds by UPLC

The extraction of the samples for phenolic compounds and their chromatographic analysis were performed exactly as described by Oszmiański and Lachowicz (2016). The samples were analyzed by an Ultra-Performance Liquid Chromatography Photodiode Array Detector (UPLC-PDA; Acquity UPLC System, Waters Corp., Milford, MA, USA). The study identified phenolic acids and their sums were calculated as chlorogenic acid, which is based on dominant compounds and compared with reference standards (Figure S1). All results were taken in triplicate and shown as mg/100 g dm (dry mass) of sample.

### 3.4. Physical Parameters

#### 3.4.1. Color Parameters by CIE Lab System 

The color properties (L *, a *, b *) of prepared products were determined by reflectance measurement with a Color Quest XE HunterLab colorimeter (Biosens, Warsaw, Poland). The samples were filled in a 1-cm cell, and L*, a*, b* values were determined using Illuminant D65 and 10° observer angle. Samples were measured against a white ceramic reference plate (L * = 93.92; a * = 1.03; b * = 0.52). The total change in color of powders (DE *) [34] and also EP, and Dom WL were measured. The data were the mean of three measurements.

#### 3.4.2. Water Activity 

Water activity (a_w_) was measured in triplicate (*n* = 3) at 25 °C ± 2 using an AQUA LAB DewPoint water activity meter (Pullman, WA, USA) [26].

### 3.5. Pro-Helathy Properties

#### 3.5.1. The Antioxidants Activity

The extraction procedure of the radical activity (ABTS), reducing potency (FRAP), and the oxygen radical absorbance capacity (ORAC) test was the same for all determinations and was carried out identically, as described by Lachowicz, Świeca, and Pejcz [35], and Nowicka, Wojdyło, Laskowski [36]. The FRAP, ABTS, and ORAC tests were prepared as previously described by Benzie and Strain [37], Re et al. [38], and Ou et al. [39], respectively. The antioxidant capacity was expressed as millimoles of Trolox per 100 g of sample. The ORAC assay was carried out on an RF-5301 PC spectrofluorometer (Shimadzu, Kyoto, Japan). Measurements by means of ABTS, and FRAP method involved a UV-2401 PC spectrophotometer (Shimadzu, Kyoto, Japan).

#### 3.5.2. Activity of α-Amylase, α-Glucosidase, Pancreatic Lipase Inhibitors

The α-amylase and α-glucosidase inhibitory effect of the sample extracts was assayed according to the procedure described previously by Nowicka, Wojdyło, Samoticha [36] while the inhibition of lipase activity was determined according to Podsędek et al. [40], respectively. Acarbose was included in the case of α-amylase and α-glucosidase as a positive control, while the orlistat was used as a positive control for pancreatic lipase. The results were expressed as IC_50_ value.

### 3.6. Statistics Methods

Statistical analysis was conducted using Statistica version 13 (StatSoft, Krakow, Poland). Significant differences (*p* ≤ 0.05) between means were evaluated by two-way ANOVA and Tuckey’s multiple range test. All data included in this study are presented as the mean value ± standard deviation and were performed at least three times.

## 4. Conclusions

The analyses of the content of polyphenolic compounds and the health-promoting activity of selected purée variants showed that the best results were obtained in JA dried with FD. It has been proved that not only the drying method but also the type of raw material used for drying played a significant role in modulating the antioxidant, anti-diabetic, and anti-obesity properties of the tested material. Hence, much better effects regarding the contents of pectin and health-promoting compounds as well as modeling health-promoting properties were obtained by drying the raw rather than the previously cooked material. The best effects of preserving the natural light color after production were obtained in the freeze-dried samples; however, due to the high costs of this method, it can be replaced by vacuum drying.

## Figures and Tables

**Table 1:** The results of analyzes of purées and dried JA.

Type of Analysis	FDC	FDR	SDC	SDR	VDC	VDR
Dry matter (g/100 g)	98.88 ± 0.20 a^a^	98.88 ± 0.20 a	97.14 ± 0.19 b	92.66 ± 0.19 e	95.90 ± 0.19 c	95.60 ± 0.19 d
Water activity (a_w_)	0.01 ± 0.00 c	0.02 ± 0.00 c	0.15 ± 0.00 c	0.36 ± 0.00 a	0.19 ± 0.00 ab	0.11 ± 0.00 c
Ash (g/100 g)	4.28 ± 0.01 a	4.18 ± 0.01 a	4.10 ± 0.01 b	3.74 ± 0.01 c	3.88 ± 0.01 c	4.16 ± 0.01 b
pH	5.86 ± 0.01 ab	5.83 ± 0.01 ab	5.77 ± 0.01 ab	5.68 ± 0.01 b	5.80 ± 0.01 ab	5.90 ± 0.01 a
Total acidity (g/100 g)	1.08 ± 0.00 a	1.09 ± 0.00 a	1.07 ± 0.00 a	1.06 ± 0.00 a	1.09 ± 0.00 a	1.04 ± 0.00 a
Pectins (g/100 g)	3.10 ± 0.01 d	4.33 ± 0.01 a	1.44 ± 0.00 f	2.46 ± 0.01 e	3.60 ± 0.01 c	4.09 ± 0.01 b
Inulin (g/100 g)	40.08 ± 0.08 e	43.32 ± 0.09 a	43.06 ± 0.09 b	41.22 ± 0.08 c	40.94 ± 0.08 d	38.94 ± 0.08 f
Fructose (g/100 g)	0.10 ± 0.00 b	0.14 ± 0.00 b	0.12 ± 0.00 b	0.40 ± 0.00 a	0.09 ± 0.00 b	0.40 ± 0.00 a
Sucrose (g/100 g)	1.33 ± 0.00 d	1.84 ± 0.00 b	1.23 ± 0.00 d	1.61 ± 0.00 c	1.55 ± 0.00 c	2.06 ± 0.00 a

^a^ Values are means ± standard deviation, *n* = 3. Mean values within a row with different letters as a, b, c, d, e, f are significantly different at *p* < 0.05. Abbreviations: FDC, freeze drying of cooked material; FDR, freeze drying of raw material; SDC, sublimation drying of cooked material; SDR, freeze drying of raw material; VDC, vacuum drying of cooked material; VDR, vacuum drying of raw material.

**Table 2:** Analysis results of phenolic compounds (mg/100 g dm) in dry JA samples.

Compounds	MS/MS	R.t. (min)	FDC	FDR	SDC	SDR	VDC	VDR
Hydroxyferulic acid hexoside ^a^	371/353/209	3.01	2.07 ± 0.00 a ^e^	1.76 ± 0.00 b	1.36 ± 0.01 c	1.47 ± 0.01 c	1.85 ± 0.00 b	1.68 ± 0.01 b
Caffeoylquinic acid ^b^ (isomer of chlorogenic acid)	353/191/179/135	3.31	33.25 ± 0.07 a	20.90 ± 0.04 c	20.46 ± 0.04 d	1.85 ± 0.00 f	28.39 ± 0.06 b	16.27 ± 0.03 e
Hydroxyferulic acid hexoside(isommer) ^a^	371/353/209	3.50	2.75 ± 0.01 b	2.28 ± 0.00 c	2.31 ± 0.01 c	5.69 ± 0.01 a	2.74 ± 0.01 b	5.61 ± 0.01 a
Hydroxyferulic acid hexoside(isommer) ^a^	371/353/209	3.66	0.40 ± 0.00 b	0.40 ± 0.00 b	0.27 ± 0.00 b	0.87 ± 0.00 a	0.35 ± 0.00 b	0.84 ± 0.00 a
Caffeoylquinic acid-quinon sulfite ^b^	415/387/258//191/179/161	3.83	nd	87.36 ± 0.17 c	nd	99.81 ± 0.20 a	nd	98.14 ± 0.20 b
Caffeoylquinic acid ^b^	353/191/179/135	3.87	2.52 ± 0.01 f	76.77 ± 0.15 c	3.47 ± 0.01 d	87.71 ± 0.18 a	2.85 ± 0.01 e	86.24 ± 0.17 b
caffeoyl-gucoside ^c^	341/179/135	4.25	7.71 ± 0.02 f	8.63 ± 0.02 e	10.98 ± 0.02 b	10.19 ± 0.02 c	8.87 ± 0.02 d	11.37 ± 0.02 a
3-*O*-Caffeoylquinic acid ^d^	353/191/135	4.36	83.20 ± 0.17 d	150.46 ± 0.30 a	51.46 ± 0.10 f	111.94 ± 0.22 c	72.38 ± 0.14 e	119.73 ± 0.24 b
Caffeoylquinic acid ^b^	353/191/179/173/135	4.51	38.54 ± 0.08 a	11.73 ± 0.02 f	26.95 ± 0.05 c	14.21 ± 0.03 d	33.02 ± 0.07 b	13.34 ± 0.03 e
Caffeoylquinic acid ^b^	353/191/179/173/135	4.79	14.19 ± 0.03 c	6.19 ± 0.01 d	14.26 ± 0.03 c	20.25 ± 0.04 b	14.31 ± 0.03 c	37.94 ± 0.08 a
Caffeoylquinic acid ^b^	353/191/179/173/135	5.13	27.31 ± 0.05 e	91.61 ± 0.18 a	23.44 ± 0.05 f	71.29 ± 0.14 c	27.63 ± 0.06 d	75.10 ± 0.15 b
Caffeoyl glucopyranose ^c^	341/179/135	5.33	4.42 ± 0.01 d	8.88 ± 0.02 c	3.02 ± 0.01 f	12.74 ± 0.03 a	3.50 ± 0.01 e	10.47 ± 0.02 b
Dicaffeoylquinic acid ^b^	515/353/191/179/173/161	5.58	13.96 ± 0.03 b	53.89 ± 0.11 a	10.12 ± 0.02 e	11.38 ± 0.02 d	12.52 ± 0.03 c	7.90 ± 0.02 f
Dicaffeoylquinic acids- quinon sulfite ^b^	577/415/387/258//191/179	5.75	nd	99.58 ± 0.20 a	nd	77.63 ± 0.16 c	nd	83.43 ± 0.17 b
Caffeoylquinic acid ^b^	353/191/179/173/161/135	6.14	9.36 ± 0.02 d	17.15 ± 0.03 c	4.86 ± 0.01 f	21.25 ± 0.04 a	7.33 ± 0.01 e	17.84 ± 0.04 b
Caffeoylquinic acid-quinon sulfite ^b^	415/387/258//191/179/161	6.25	nd	12.89 ± 0.03 a	nd	7.78 ± 0.02 b	Nd	6.04 ± 0.01 c
Hydroxyferulic acid hexoside (isomer 3) ^a^	371/353/209	6.33	2.05 ± 0.01 d	4.47 ± 0.01 b	1.44 ± 0.01 f	4.88 ± 0.01 a	1.77 ± 0.01 e	3.36 ± 0.01 c
Hydroxyferulic acid hexoside (isomer 3) ^a^	371/353/209	6.57	4.02 ± 0.01 b	4.70 ± 0.01 a	2.38 ± 0.01 e	2.84 ± 0.01 d	3.34 ± 0.01 c	1.80 ± 0.01 f
Hydroxyferulic acid hexoside (isomer 3) ^a^	371/353/209	6.96	1.37 ± 0.00 d	26.36 ± 0.05 a	0.95 ± 0.00 e	24.90 ± 0.05 b	1.11 ± 0.00 e	19.28 ± 0.04 c
Dicaffeoylquinic acids- quinon sulfite ^b^	577/415/387/258//191/179	7.04	nd	75.95 ± 0.15 a	nd	71.74 ± 0.14 b	Nd	55.55 ± 0.11 c
3,4-Di-*O*-caffeoylquinic acid ^b^	515/353/191	7.51	36.42 ± 0.07 a	24.15 ± 0.05 c	20.28 ± 0.04 d	19.23 ± 0.04 e	31.14 ± 0.06 b	12.88 ± 0.03 f
3,5-Di-*O*-caffeoylquinic acid) ^b^	515/353/191	7.77	34.89 ± 0.07 d	118.37 ± 0.24 a	17.69 ± 0.04 f	68.39 ± 0.14 c	28.66 ± 0.06 e	77.14 ± 0.15 b
Hydroxyferulic acid hexoside (isomer 3) ^a^	371/353/209	7.94	1.38 ± 0.00 d	2.64 ± 0.01 a	0.77 ± 0.00 e	1.75 ± 0.00 c	0.90 ± 0.00 e	1.97 ± 0.00 b
1,5-Di-*O*-caffeoylquinic acid ^b^	515/353/191	8.21	32.40 ± 0.06 a	14.80 ± 0.03 d	17.26 ± 0.03 c	11.24 ± 0.02 e	27.39 ± 0.05 b	9.37 ± 0.02 g
Hydroxyferulic acid hexoside (isomer 3) ^a^	371/353/209	8.48	0.83 ± 0.00 d	1.58 ± 0.01 a	0.40 ± 0.00 f	1.14 ± 0.00 b	0.63 ± 0.00 e	0.99 ± 0.00 bc
Sum of phenolic acids			353.05 ± 19.87 d	923.53 ± 43.70 a	234.13 ± 12.35 f	762.16 ± 35.70 b	310.68 ± 17.23 e	774.28 ± 36.67 c

^a^ The calibration curve of ferulic acid was used to quantify; ^b^ the calibration curve of 5-*O*-caffeoylquinic was used to quantify; ^c^ the calibration curve of caffeic acid was used to quantify; ^d^ the calibration curve of 3-*O*-caffeoylquinic acid was used to quantify; ^e^ values are means ± standard deviation, *n* = 3. Mean values within a row with different letters as a, b, c, d, e, f are significantly different at *p* < 0.05. Abbreviations: FDC, freeze drying of cooked material; FDR, freeze drying of raw material; SDC, sublimation drying of cooked material; SDR, freeze drying of raw material; VDC, vacuum drying of cooked material; VDR, vacuum drying of raw material; R.t., retention time; nd, not detected.

**Table 3:** Color measurement results.

Type of Sample	L *	a *	b *
FDR	92.51 ± 0.19 a ^a^	–1.22 ± 0.01 e	12.11 ± 0.02 c
FDC	90.22 ± 0.18 b	–1.45 ± 0.01 f	10.88 ± 0.02 e
SDR	88.32 ± 0.18 c	–0.40 ± 0.00 b	14.10 ± 0.03 b
SDC	85.76 ± 0.17 e	–0.94 ± 0.00 d	11.67 ± 0.02 d
VDR	84.63 ± 0.17 f	–0.79 ± 0.00 c	14.05 ± 0.03 b
VDC	86.99 ± 0.17 d	–0.26 ± 0.00 a	14.59 ± 0.03 a

^a^ Values are means ± standard deviation, *n* = 3. Mean values within a row with different letters as a, b, c, d, e, f are significantly different at *p* < 0.05. Abbreviations: FDC, freeze drying of cooked material; FDR, freeze drying of raw material; SDC, sublimation drying of cooked material; SDR, freeze drying of raw material; VDC, vacuum drying of cooked material; VDR, vacuum drying of raw material.

**Table 4:** The degree of metabolism and absorption of sugars derived from JA products.

Type of Sample	α-Glucosidase	α-Amylase	Pancreatic Lipase	ABTS	FRAP
	IC_50_ (mL/mL)	IC_50_ (ug/mL)	IC_50_ (mL/mL)	(mmol TE/100 g dm)	(mmol TE/100 g dm)
FDR	166.70 ± 0.33 e ^a^	177.98 ± 0.36 c	43.75 ± 0.09 c	3.73 ± 0.01 d	1.69 ± 0.01 e
FDC	193.29 ± 0.39 f	297.90 ± 0.60 f	45.86 ± 0.09 e	7.65 ± 0.02 a	2.67 ± 0.01 a
SDR	123.60 ± 0.25 b	136.98 ± 0.27 b	31.48 ± 0.06 a	2.78 ± 0.01 f	1.46 ± 0.00 f
SDC	135.52 ± 0.27 c	185.87 ± 0.37 d	58.34 ± 0.12 f	6.91 ± 0.01 c	2.60 ± 0.01 b
VDR	150.94 ± 0.30 d	221.19 ± 0.44 e	45.06 ± 0.09 d	2.85 ± 0.01 e	1.97 ± 0.01 d
VDC	120.31 ± 0.24 a	129.97 ± 0.26 a	33.08 ± 0.07 b	7.09 ± 0.01 b	2.14 ± 0.01 c

^a^ Values are means ± standard deviation, *n* = 3. Mean values within a row with different letters as a, b, c, d, e, f, are significantly different at *p* < 0.05. Abbreviations: FDC, freeze drying of cooked material; FDR, freeze drying of raw material; SDC, sublimation drying of cooked material; SDR, freeze drying of raw material; VDC, vacuum drying of cooked material; VDR, vacuum drying of raw material.

## Data Availability

The authors declare that the data is available.

## Supplementary Materials

Supplementary Materials Figure S1: chromatograms registered at 280 nm of dried purée from Jerusalem artichoke: with derivatives of SO_2_ (1), without derivatives of SO_2_ (2).

## Sample Availability

Samples of the compounds as JA are available from the authors.
