# Supplementary material for: Evaluation of Innovative Dried Purée from Jerusalem Artichoke—In Vitro Studies of Its Physicochemical and Health-Promoting Properties
Source: Molecules. 2021 Apr 30;26(9):2644. doi: 10.3390/molecules26092644 (PMC8125012; doi:10.3390/molecules26092644)
Supplement: Supplementary file 1 [file molecules-26-02644-s001.zip › molecules-1192996-supplementary.pdf]

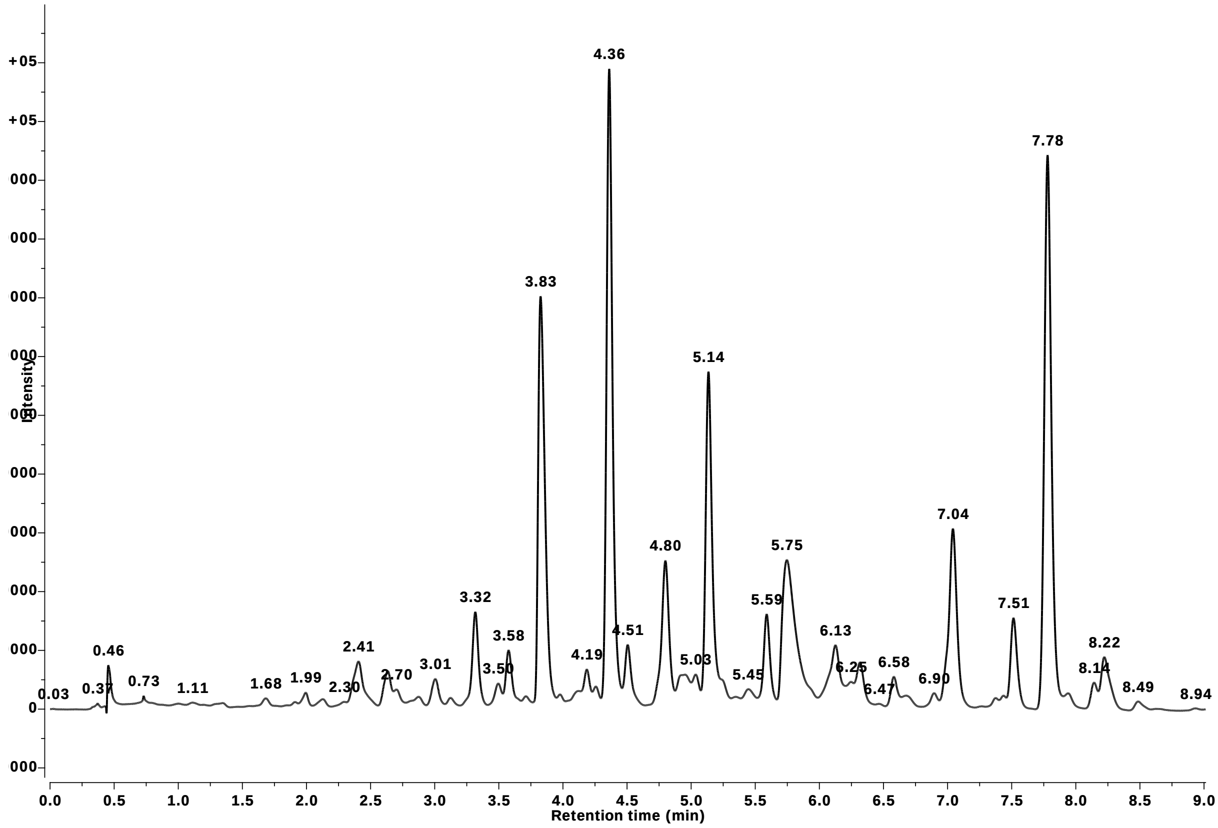

Intensity
Retention time (min)
4.36
7.78
3.83
5.14
7.04
4.80
5.75
3.32
5.59
7.51
3.58
4.51
6.13
8.22
0.46
2.41
4.19
5.03
3.50
6.25
6.58
8.14
2.70
3.01
5.45
6.90
0.73
1.11
1.68
1.99
2.30
6.47
8.49
0.03
0.37
8.94

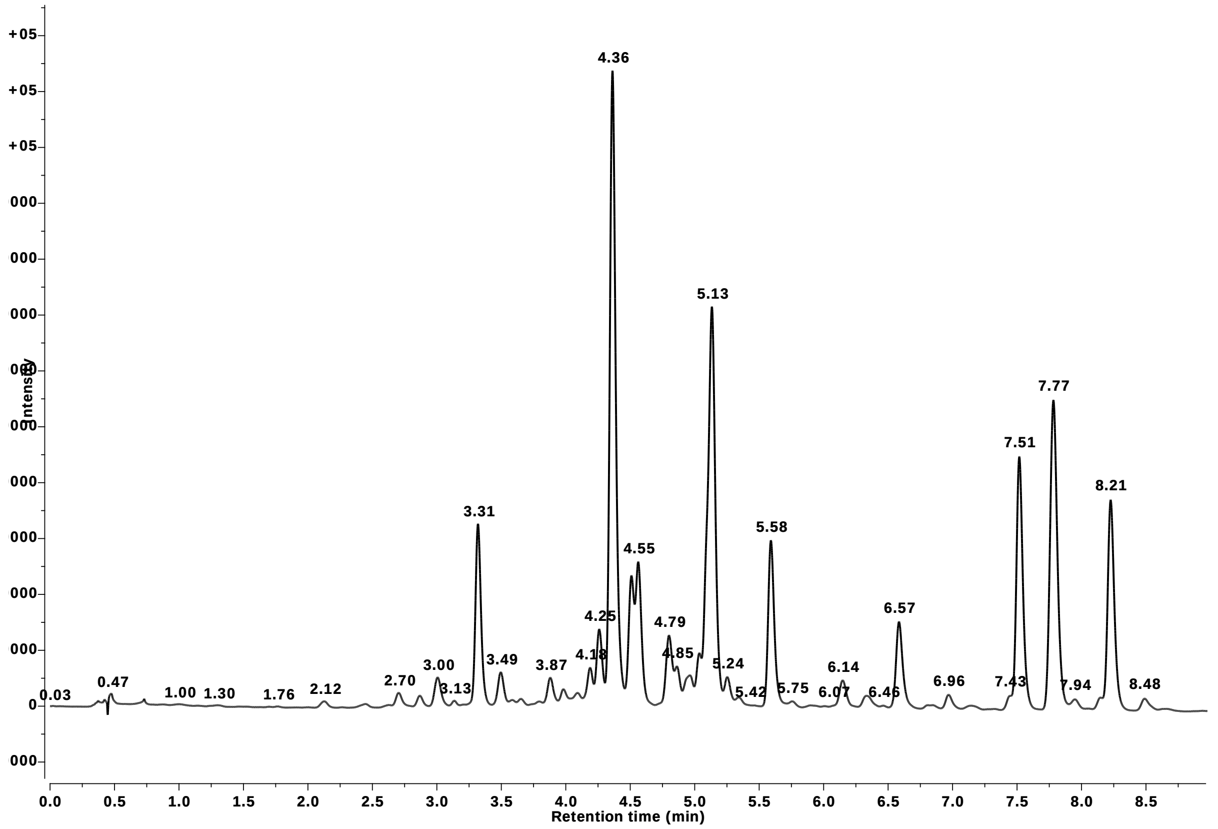

Intensity
Retention time (min)
4.36
5.13
7.77
7.51
8.21
3.31
5.58
4.55
6.57
4.25
4.79
4.18
4.85
5.24
3.00
3.49
3.87
6.14
0.47
2.70
3.13
6.96
7.43
7.94
8.48
1.00
1.30
1.76
2.12
5.42
5.75
6.07
6.46
0.03

Supplementary Materials S1. Chromatograms registered at 280 nm of dried purée from Jerusalem artichoke: with derivatives of SO_2 (1), without derivatives of SO_2 (2).
